# Louis Jacobsohn-Lask (1863–1940)

**DOI:** 10.1007/s00415-024-12260-0

**Published:** 2024-03-06

**Authors:** Ibrahim Demircubuk, Esra Candar, Gulgun Sengul

**Affiliations:** 1 Department of Anatomy, Institute of Health Sciences, Ege University, Izmir, Turkey; 2 Department of Neuroscience, Institute of Health Sciences, Ege University, Izmir, Turkey; 3 School of Medicine, Department of Anatomy, Ege University, Izmir, Turkey

**Keywords:** Bekhterev-Jacobsohn reflex, Brainstem, Comparative anatomy, Jacobsohn-Lask, Spinal cord

The physician and neuroanatomist Louis Jacobsohn-Lask (Fig. 1) was born on March 2, 1863, in Bromberg (then in the Prussian Province of Posen, today Bydgoszcz in Poland). His Jewish family moved to Berlin in the 1870s to escape antisemitism. He obtained his medical degree in 1888 from Friedrich Wilhelm University in Berlin and earned his doctorate in 1889 at the Second Medical Clinic of Charité Hospital. After a short period of private practice, he trained under the neuropsychiatrist Emanuel Mendel, founder of *Neurologisches Centralblatt*, and the anatomist Wilhelm von Waldeyer-Hartz. Thanks to Mendel, Jacobsohn had the opportunity to meet renowned physicians in Berlin, including the neurologist Edward Flatau, the ophthalmologist Bernhard Pollack, and the neuropathologist Siegfried Kalischer [1].

**Fig. 1:**
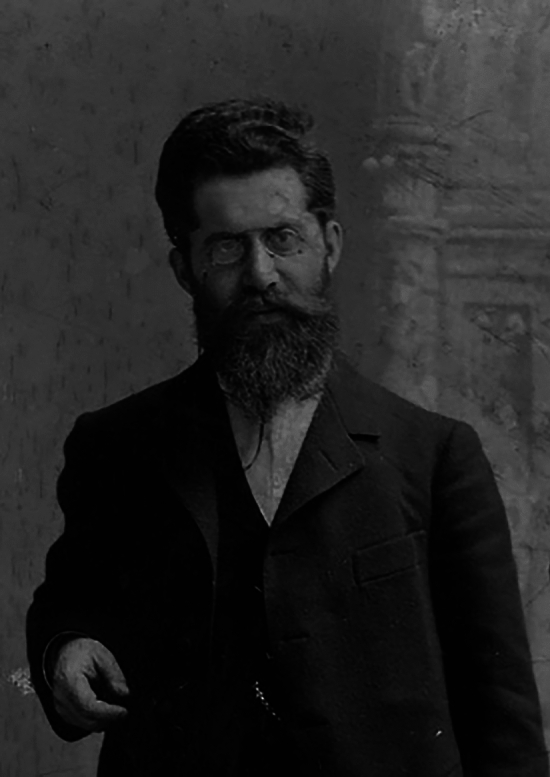
Louis Jacobsohn-Lask (1863–1940). Courtesy of Wikimedia Commons; image outlined by Eisenberg [2], derived from the Lask family archives

In 1899, Jacobsohn collaborated with Flatau, one of the founders of modern Polish neurology. They published the “Handbook of anatomy and comparative anatomy of the central nervous system of mammals” [3]. They noticed the incomplete and scattered knowledge of brain and spinal cord regions in previous anatomical textbooks and articles. They described the central nervous system of many mammals, including the chimpanzee, orangutan, rhesus macaque, cat and dog. They illustrated many mammalian brains, summarizing their findings in tables. In 1908, the *“*Handbook of the pathological anatomy of the nervous system”, edited by Flatau, Jacobsohn and Flatau’s Moscow teacher Lazar Solomonovich Minor was published, where they used notable terms for spinal cord anatomy [4]. With over 200 figures, this book focused on the pathological anatomy of nervous diseases, including poliomyelitis, multiple sclerosis, and developmental malformations of the spinal cord. Jacobsohn collaborated with the biochemist Leonor Michaelis to describe methods of investigating the nervous system in the first chapter of the book.

Jacobsohn employed a special method for studying the spinal cord, whereby he treated a fresh adult male spinal cord with 96% alcohol for 24 h. Tissue block sections were embedded in paraffin, cut into 10–20 µm thick sections, stained with toluidine blue, and examined under the microscope after immersion in Canadian balsam. In his book “On the nuclei of the human spinal cord”, Jacobsohn acknowledged Benedict Stilling, Jacob Augustus Lockhart Clarke, and Waldeyer for their descriptions of nerve cells in the grey matter of the spinal cord [5]. He introduced significant terms related to spinal cord anatomy in this publication.

The Swedish neuroscientist Bror Anders Rexed, in his 1952 article, divided the spinal cord into distinct laminae based on cellular characteristics and functional organization. When comparing the terminologies used by Jacobsohn-Lask and Rexed, it becomes apparent that there is correspondence between them. For example, Jacobsohn-Lask’s “nucleus magnocellularis pericornualis” corresponds to Rexed’s lamina I, “nucleus sensibilis proprius” corresponds to lamina II, and “nucleus magnocellularis centralis cornu posterioris” corresponds to laminae III-IV [6]. This demonstrates the consistency and relevance of Jacobsohn-Lask’s terminology in relation to the later classification by Rexed. Jacobsohn used the term “nucleus magnocellularis basalis s. spino-cerebellaris”, which is now known as the “dorsal nucleus” or “Clarke nucleus”. He expressed a preference for this nomenclature because of the fiber system projecting from these cells to the cerebellum. Additionally, in one of his figures depicting the S4 segment, he demonstrated nuclei referred to as “nuclei sympathici sacro-coccygeales”, which partially corresponds to what is now known as the “sacral precerebellar nucleus” or “Stilling sacral nucleus.” In the same year, Jacobsohn published an article *“*On the finger flexion reflex” [7], where he defined a reflex similar to that described by Vladimir Bekhterev in 1903. This reflex, observed in pyramidal disorders, is characterized by extension and abduction of the thumb when pressure is applied distally on the radial edge of the forearm. It is also referred to as the Bekhterev–Jacobsohn reflex [8, 9].

In 1909, Jacobsohn published his book “On the nuclei of the human brainstem” [10]. He employed a similar method to the one he used for the spinal cord, but this time he sectioned the brainstem at a thickness of 20–30 µm. The book, consisting of 70 pages and 27 figures, presented remarkable information about brainstem nuclei. Jacobsohn displayed these nuclei in cross-sections spanning from the decussation of the pyramids to the thalamic region. Jacobsohn noted that nerve cells in the brainstem are more clustered than those in the spinal cord. He divided brainstem nuclei into nine groups based on their functions: end motor nuclei, superordinate motor nuclei, motor-sympathetic nuclei, specialized motor nucleus, sensitive nuclei, sensitive pigment nuclei, distal sensory nuclei, superordinate sensory nuclei, and cerebellum-related nuclei. Instead of using the names of the researchers who first defined these nuclei, Jacobsohn preferred to name them according to their function, location, and cellular characteristics. He also introduced the term “nucleus tegmenti pedunculo-pontinus” and depicted this nucleus in cross-sections; it corresponds to the present-day “pedunculotegmental nucleus.”

In 1919, Jacobsohn added to his last name that of his wife, the German playwriter Berta Lask (1878–1967), and thus became Louis Jacobsohn-Lask. Despite his fundamental contributions to science, he was never promoted to the higher academic ranks, other than that of an unsalaried lecturer (titular professor), because of his Jewish ancestry. Until the National Socialists came into power in 1933, Jacobsohn-Lask taught neurology and neuroanatomy at the University of Berlin. However, due to the growing antisemitic climate, he reluctantly decided to emigrate to the Soviet Union in 1936 at the age of 73 years. He settled in Sevastopol, where he lived until his passing on May 17, 1940. Jacobsohn-Lask contributed pivotal works to the fields of neurology, human and comparative neuroanatomy. Despite the challenges and discrimination that he confronted, he can be regarded as one of the prominent neuroanatomists of his era. In recognition of his accomplishments, a ceremonial funeral was held in Sevastopol to honor his memory [1].

## Data Availability

Not applicable.
